# Perspectives and Views of Primary Care Professionals Regarding DiabeText, a New mHealth Intervention to Support Adherence to Antidiabetic Medication in Spain: A Qualitative Study

**DOI:** 10.3390/ijerph19074237

**Published:** 2022-04-01

**Authors:** Rocío Zamanillo-Campos, Maria Jesús Serrano-Ripoll, Joana Maria Taltavull-Aparicio, Elena Gervilla-García, Joana Ripoll, Maria Antonia Fiol-deRoque, Anne-Marie Boylan, Ignacio Ricci-Cabello

**Affiliations:** 1 Research Group in Primary Care and Promotion—Balearic Islands Community (GRAPP-caIB), Health Research Institute of the Balearic Islands (IdISBa), 07120 Palma de Mallorca, Spain; mariajesus.serranoripoll@ibsalut.es (M.J.S.-R.); joanamaria.taltavull@ibsalut.es (J.M.T.-A.); joana.ripoll@ibsalut.es (J.R.); ignacio.ricci@ibsalut.es (I.R.-C.); 2 Primary Care Research Unit of Mallorca (IB-Salut), Balearic Health Service, 07002 Palma de Mallorca, Spain; 3 Primary Care Preventive and Health Promotion Research Network (redIAPP), 08007 Barcelona, Spain; 4 Psychology Department, University of the Balearic Islands (UIB), 07120 Palma de Mallorca, Spain; elena.gervilla@uib.es; 5 Statistical and Psychometric Procedures Applied in Health Science, Health Research Institute of the Balearic Islands (IdISBa), 07120 Palma de Mallorca, Spain; 6 Nuffield Department of Primary Care Health Sciences, University of Oxford, Oxford OX2 6GG, UK; anne-marie.boylan@phc.ox.ac.uk; 7 Biomedical Research Center in Epidemiology and Public Health (CIBERESP), 28029 Madrid, Spain

**Keywords:** type 2 diabetes, SMS, self-care, medication adherence, mobile health, eHealth, qualitative research

## Abstract

Background: Antidiabetic medication is effective in preventing diabetes-related complications. However, 40% of type 2 diabetic patients do not adhere to their medication regimes adequately. Brief text messages represent a promising approach to support medication adherence. The aim of this study was to explore the perspectives of primary care professionals (PCPs) concerning the DiabeText intervention, a new text messaging intervention to be developed to support medication adherence in people with type 2 diabetes (T2D) in Mallorca, Spain. Methods: We conducted four focus groups (*n* = 28) and eight semi-structured interviews with doctors and nurses. Data collection and analysis were carried out by researchers independently following Braun and Clark’s methodology. Results: Three main themes were identified: (1) text messaging interventions have the potential to effectively support diabetes self-management; (2) involving PCPs in the intervention would facilitate its design and implementation; (3) obtaining evidence supporting the cost-effectiveness is a key prerequisite for large-scale implementation of the intervention. PCPs identified barriers and enablers of the design and implementation of the intervention and made suggestions about the content and format of the text messages. Conclusion: The DiabeText intervention is perceived as useful and acceptable by PCPs provided its cost-effectiveness.

## 1. Introduction

Type 2 diabetes (T2D) is a common, life-long condition affecting 9.3% of people worldwide [1]. Alongside lifestyle changes, medicines are used to lower blood glucose, blood pressure, and lipids to prevent long-term complications such as cardiovascular disease, retinopathy, neuropathy, and nephropathy. International studies show that up to 37% of diabetes patients stop their blood glucose-lowering medicine within one year of starting treatment [2]. Adherence falls further as the number of tablets increases [3]. For those who continue with treatment, about 70–80% of doses are taken as prescribed [4].

A recent Cochrane systematic review concluded that despite an understanding of the complexity of adherence behavior, current interventions for improving medicine use in long-term conditions are not very effective [5]. Mobile Health (mHealth) interventions, currently widely used for healthcare delivery [6], constitute a promising strategy to support medication adherence [7]. They have the potential to address a range of different problems that might lead to non-adherence, including advice about the practical issues of taking tablets and tablet collection, providing information about medicine, giving advice about setting up routines, and prompting contact with a health professional where there are concerns. Recent trials show that automated brief text messages (e.g., SMS), which are delivered at a wide scale and low cost via digital health systems and added to usual care, can be effective in supporting T2D prevention [8] and in improving T2D risk factors such as overweight [9], hypertension [10], hyperlipidemia [11], and smoking behavior [12].

In Spain, non-adherence rates to oral antidiabetic drugs are particularly high, ranging from 45% to 52% [13,14,15,16,17,18]. In addition, Spain is the country with the highest mobile phone usage rates for adults in the world at 99% [19]. Therefore, an intervention based on the use of automated brief text messages to support medication adherence has the potential to reach a very large proportion of the Spanish population. However, to date, no text messaging intervention to support medication adherence has been developed in Spain. In this context, and as part of a four-year nationally-funded program of work, we set out to develop DiabeText, an intervention based on the use of a mobile-device system delivering automated, tailored brief messages to support medication adherence in people with T2D. Best practice guidelines [20] highlight the need to take into account the perspectives of relevant stakeholders as part of the design of complex interventions. Formative work exploring the views of patients regarding the DiabeText intervention has been previously published [21]. Formative qualitative research exploring the views of healthcare professionals is a key prerequisite; to ensure the intervention is well aligned with routine clinical practice; to identify contextual factors and unexpected mechanisms, by which the intervention may or may not produce its intended benefits; and to identify potential implementation barriers.

The aim of this study was to explore the views and perspectives of primary care professionals (PCPs) concerning the DiabeText intervention, a new text messaging intervention to be developed to support medication adherence in people with T2D in Mallorca, Spain. The specific objectives were: (1) to explore the barriers and enablers of the implementation of a new technological service to improve T2D management in the primary care system of the Balearic Islands; (2) to identify key components to inform the intervention design.

## 2. Materials and Methods

### 2.1. Participants

This qualitative study involved eight individual semi-structured interviews and four focus groups with 28 PCPs. One focus group included six family doctors and one nurse; one group included three doctors and four nurses; and the remaining two groups each included four doctors and three nurses. Individual interviews included two primary care doctors, three primary care nurses, one community pharmacist, one health decision maker, and one hospital endocrinologist. The inclusion of participants was based on their previous experience in the provision of healthcare to people with T2D. Sampling was purposive according to the professional category (nurses and doctors) and level of expertise in diabetes management. We invited all the PCPs through the manager of their health center and every PCP who accepted to take part in the study was included. Focus group participants were homogeneous in the sense that each focus group was held with PCPs from the same center, therefore using the same clinical protocols and taking care of the same patient population. Participants were recruited through research collaborators and coordinators from PC centers. Before all the interviews and focus groups, participants received patient information sheets and signed informed consent forms. This study was approved by the Research Ethics Committee of the Balearic Islands (CEI-IB) in July 2019 (39/48/19 PI).

### 2.2. Data Collection

Data collection took place between October 2019 and January 2020. Semi-structured interviews were conducted face-to-face or telephonically, audio-recorded, and lasted 15–50 min. All of the focus groups were held face-to-face in PC centers. The sessions, which lasted approximately 60–90 min, were video-recorded to ensure accuracy. The duration of the interviews was generally shorter than that of the focus groups because during the focus groups multiple points of view were elicited, and participants were encouraged to interact among themselves. Focus groups and interviews were employed in an attempt to gain the broadest range of views from the participants. The focus groups were used to explore how primary care staff talked about the topic together and to determine the main issues from their perspectives. The interviews complemented this approach by allowing us to explore these issues in more depth with individuals. Interviews have the added benefit of allowing participants to express in private views that they may not wish to present to other colleagues. So, in using both approaches, we ensured that we captured a broader range of in-depth data.

Interviews and focus groups were facilitated by a member of the research team, with another researcher acting as an observer and taking field notes. To minimize the impact of the researchers on the data collection, all researchers engaged in a reflexivity exercise, reflecting on their professional role and their assumptions about the intervention before they interviewed participants. We used the same topic guide (Box 1) for the individual interviews and the focus groups. The topic guide was developed before the commencement of the study based on the statement of the specific objectives of the study and with the support of a senior qualitative researcher from our group.

Box 1 **Focus group guide that was developed ad hoc by the team based on the objectives of the study and bibliography consultation.**

ACCEPTABILITY AND PERCEIVED USEFULNESS OF THE DIABETEXT INTERVENTION
In general terms, what do you think of the idea of sending SMS to patients’ mobile phones with information to improve diabetes care?
What impact do you think these messages could have on the patients?
How useful do you think this type of intervention is?
Do you think that this intervention is a good opportunity or do you perceive any limitations?
What content do you think should be included in these messages?
What features do you think the SMS should be customized by?

ENABLERS AND BARRIERS TO THE IMPLEMENTATION OF THE DIABETEXT INTERVENTION
What possible impact could it have to roll out this new service as part of the Balearic Islands Health Service portfolio?
What barriers or difficulties do you anticipate could arise from the implementation of this service?
What possible facilitating elements would you identify for the implementation of this type of intervention?
How do you think this service could interfere with your usual clinical practice?
Do you have any suggestions for the health system to promote in some way the potential beneficial effects of this intervention?
Do you have any other comments or suggestions that you think we should take into account when designing this messaging service?

The topics included the acceptability and usefulness of an mHealth intervention to support diabetes self-management (DSM) and the barriers and enablers to mHealth development and implementation. Following established methods for qualitative research, the guide was used to steer the data-collection process, rather than to dictate it. During the focus groups, co-facilitators (observers) took notes, paying particular attention to nonverbal communication. At the end of each focus group, the facilitator and the observers met for around 20 min to debrief. Comments focused on initial reflections on the data, exploration of first impressions emerging from the group, and discussions about the main ideas exposed by the participants. Focus groups and interviews continued until data saturation was reached, i.e., until preliminary analysis indicated that strong patterns were becoming apparent in data collection and nothing new was being discussed. We registered the characteristics (age, gender, and professional role) of the participants in the focus groups and individual interviews. Recordings were transcribed verbatim by professional typists. The transcripts were read by the first author and cross-checked with the audiotapes or the videotapes to ensure the accuracy of the transcriptions.

### 2.3. Data Analysis

Before starting the data analysis, the research group shared their backgrounds and main preconceived ideas to ensure their interests were not intruding on the data-analysis process. A thematic analysis approach was then used to analyze the data. Based on Braun and Clark’s methodology [22], our analysis involved the following stages: First, all data were coded by the lead author using an iterative approach after immersion in the data. Initial notes were made, followed by a process of categorization and theme development. Second, initial themes on the acceptance and perceived utility of the SMS system by PCPs were developed and later discussed with other members of the research team, who each analyzed two transcripts by making notes, highlighting issues of importance, and developing initial themes. During the meetings, in pairs, we searched for a broader level of themes. Finally, the themes were then discussed in a final meeting with all the team members. Discrepancies were resolved by consensus. The analysis involved a constant moving back and forward between the entire data set, coded extracts of data, and the analysis itself. Writing was also part of the entire process from the very beginning through the entire analysis process. After the final meeting, we reviewed and refined themes in a series of iterations in order to define themes and write the final report.

We followed an investigator triangulation strategy [23], where data was independently analyzed by all members of the research team (except A.-M.B.) and discussed in a series of six meetings and a workshop. In addition, the data collected in the interviews and focus groups were analyzed together and triangulated (data source triangulation) to produce a more complete picture of the data than relying on either method alone. To compare and contrast between the focus groups and interviews was not the objective; rather, we aimed to triangulate to produce more holistic and in-depth findings. These two triangulation approaches allowed us to obtain greater interpretative and analytical wealth and ensured the breadth of the data was incorporated in the analysis. All researchers agreed on the final results and accepted them being representative of the data.

We included in the results section a selected number of quotations (an extended, tabulated list of quotations is available in Table S1).

## 3. Results

A total of 36 health professionals from eight primary care centers and one hospital in Mallorca agreed to participate in the study. None of the professionals we approached declined to be interviewed. The age of the participants ranged from 24 to 64 years old. Of the 36 participants, 26 (72%) were women. Participants were PC doctors (*n* = 21), PC nurses (*n* = 12), a health decision maker (*n* = 1), an endocrinologist (*n* = 1), and a community pharmacist (*n* = 1).

Three main themes were developed concerning the acceptability and utility of the proposed mHealth intervention: (1) The intervention has the potential to effectively support the provision of diabetes care. (2) Involving health professionals in the intervention would facilitate its design and implementation. (3) PCPs raised some concerns around limitations to consider during the design of the intervention.

### 3.1. The Intervention Has the Potential to Effectively Support the Provision of Diabetes Care

#### 3.1.1. DiabeText Could Increase Awareness of the Disease and Adherence to Treatment

The participants discussed how mobile technology use was widespread, that patients were used to it, and, as such, concluded that it could be an appropriate vehicle to engage patients with inadequate DSM. As one doctor said, ‘*I think we should use these technologies if we have them because people use them more and more*’ (man, 40 years). The participants felt that SMSs could raise patients’ awareness about their illness, therefore improving their self-efficacy, which is needed for diabetes self-management. As another doctor explained ‘*The messages could be used as a means to reach all those diabetic patients who are not aware that they are diabetic. For example, I have patients who take metformin, but still, they are not aware that they have diabetes*’ (woman, 46 years). SMSs could also help patients to feel supported, more cared for, and more connected with the health care system. PCPs think that monitoring and having a close relationship with patients could improve adherence to treatment.

*“I think that for us, it [the intervention] could be a positive thing, in the sense that patients would perceive that the health system takes care of them, their treatment is taken into account, and that, therefore, they are taken care of, and one way to show it is by providing reminders about how they have to take their medication when to take the pills, and perhaps additional information to reinforce adherence or lifestyles. I think that everything—taking into account the person and monitoring them—has a positive impact that helps to improve adherence. And if adherence improves, control improves, and if control improves, the system is more sustainable”.*

(Woman, 48, Nurse.)

#### 3.1.2. DiabeText Could Support Medical Consultations

The participants highlighted that they do not have the time to provide the sort of care that patients with diabetes require, which resulted in them feeling like they were losing personal connections with their patients. They discussed several reviews and tests that T2D patients require, such as diabetic foot, retinography, and renal function tests, and agreed that it was difficult to keep up with them, partly because of the complex information technology (IT) systems they used.

*“We have lots of data, indicators, but we have less and less connection with our patients. Every time we diagnose more, but we do worse at controls and follow-ups. I think the key thing is to spend time with our patients”.*

(Man, 47, Doctor.)

*“It happens to us many times: we can shake hands with the patient, but we are not able to look at their feet”.*

(Man, 47, Doctor.)

Professionals said that the intervention could reinforce the work they do with their patients, but to do that, messages should be evidence based and aligned with the current treatment provided to the patient.

*“I think that, if it were to be implemented…outside of …the study framework… obviously, the contents will have to be kept updated as time goes by. Then, primary care professionals, who are active and knowledgeable, could contribute to modifying the messages, incorporating the views of the professionals who have direct contact with patients…so that their [patients’] opinion is taken into account.”*

(Woman, 47, Doctor.)

They perceived that the system could reactivate communication between patients and PCPs about the information they are receiving through SMSs. They highlighted that sometimes, people with T2D forget to ask questions during consultations and suggested that SMSs could increase patient engagement. As one nurse pointed out, ‘*We will explain to the patient that they will receive messages to guide them through the diabetes care management and if they have any doubt, they can come to us for solving it*’ (Woman, 33 years).

It was suggested that the program could reinforce the information PCPs provide about diabetes care during the consultations. It could also provide supplemental information at times omitted during the consultations due to time constraints or competing priorities. For example, the system could serve as a reminder about the importance of following a healthy lifestyle. As one doctor said, ‘*The most difficult thing for us is to inculcate in them a change of habits, diet, and exercise*.’ (Man, 40 years).

#### 3.1.3. DiabeText Could Support the Promotion of DSM

The participants agreed that the proposed text messaging intervention could encourage patients to request specific tests after being prompted by an SMS reminder. This would empower patients and ensure they receive a higher quality of diabetes care. As one nurse said, ‘*the mHealth intervention could take away from us the paternalism role that we are playing, but we are desperate not to act like that*’ (Woman, 58 years).

*“The patients are ones who should be aware of their situation, their illness, and they should be the ones saying “I need to have my feet checked”, for example.”*

(Woman, 30, Nurse.)

PCPs suggested that a text-messaging system could have the potential to engage people to attend future appointments and to reach patients who do not have enough time to attend medical visits, thus perhaps leading to more interactions with primary care. As one doctor pointed out ‘*[the messaging intervention] is especially useful for this type of patients we usually do not reach—the ones not coming to appointments, who are the ones less well-controlled.*’ (Man, 57 years).

SMSs were also seen as potentially useful for improving patients’ health literacy. At home, in contrast to the time spent during clinical appointments, patients have the time to read SMS content quietly and the tools (such as a computer with access to the Internet) to look at extra information related to DSM. Thus, patients could learn more about their condition and improve their self-efficacy to help manage it, which may ultimately result in improving medication adherence.

*“[the messaging intervention] is good to receive it at home, where you are calm and can analyze everything the doctor told you during the consultation. Patients say “yes, yes, I understand, yes, yes”, but actually they do not understand anything.”*

(Woman, 47, Doctor.)

They perceived that improving patients’ awareness about the importance of adequate DSM could reduce the time spent in appointments, which would have the added benefits of decreasing PCPs workload and decreasing the burden on patients while improving the quality of the health care provided.

### 3.2. Involving Health Professionals in the Intervention Would Facilitate Its Design and Implementation

#### 3.2.1. PCPs Highlight the Importance of Being Part of the Project for Its Success

Participants said it was important that professionals who would be involved in prescribing the intervention felt part of it and contributed actively to its design and development. As one doctor said, ‘*Primary care professionals could shape the messages, incorporating their point of view (…). Professionals could contribute to incorporate the patients’ views on the messages, offering a means of communication so that their opinion is taken into account’* (Woman, 47 years).

To recommend the messaging program to their patients, PCPs had to be well informed about it, trust the program, and perceive it as a useful tool. In fact, some participants requested a complete list of the SMSs that were to be sent, because they wanted to know the details about the intervention, which some of the patients they attended to would be receiving, in order to be prepared for questions and to have a complete picture of the treatment.

Participants suggested that the implementation of the system should include a communication strategy targeted at PC professionals to inform them and obtain their support and warned that they needed to be involved for the intervention to succeed.

*“If we make an extraordinary product, but we do not know how to advertise it, we will not have a high market share, we will even have to close the company.”*

(Man, 47, Doctor.)

In some cases, there was some fear of feeling left out, losing control of their patients’ care, and not being at the heart of it. As one nurse said, ‘*The primary care team has to receive the recognition they deserve. The messages should not replace their work*’ (woman, 30 years). This idea contrasts with encouraging DSM and abandoning paternalism in diabetes care (see the previous section), which is presented as a limitation for the implementation of the intervention proposed in Section 3.3.1.

#### 3.2.2. PCPs Identified and Proposed the Characteristics and Content That DiabeText Should Include

The PCPs were familiar with the features and factors influencing DSM. They discussed how people with T2D are diverse, so the intervention needs to be adapted in terms of their health literacy, diabetes complications, proficiency at using a mobile phone, language barriers, culture, habits, and psychological characteristics. Accordingly, they made several suggestions concerning the content of the messages, as well as the tone, timing, and cultural adaptations (Box 2).

Box 2 **Proposed characteristics and content of SMSs to align the mHealth intervention with routine clinical practice for diabetes care.**

CONTENT:

- The secondary effects of medication; what to do when you have a fever; iodinated contrast tests; regimen changes.
- Diet, food recipes, and healthy choices when eating out.
- Avoid scaring patients with too much information about the consequences of diabetes but without taking this information out of the messages.
- Include messages about monitoring with blood test results or bodyweight goals reached
- Embed links to websites, videos, and other digital resources.
- Include reminders to take pills, pick up medication, and attend medical appointments (including retinography tests).
- Exercise, sports events, and motivation to practice sport.
- Skin and foot hygiene, and prevention of complications (such as diabetic foot, renal damage, cardiovascular diseases).
- Tobacco and alcohol abuse.
- Sensitive topics (such as sexuality or psychological concerns).
- False beliefs about diabetes.
- Traveling with diabetes and what patients should do if they forget or lose their medica-tion when traveling.
- Resources available at community pharmacies; packaging the medication by community pharmacists; solving doubts about a medication regimen or secondary effects; printing a medication dispensing calendar.

TONE:

- Behavioral change strategies should focus on increasing motivation rather than on im-posing obligations.
- Being objective and assertive, and avoiding soft communication
- Positive reinforcement: Messages should focus on health rather than on illness, enhancing the abilities and skills of people, rather than their barriers and limitations

ADAPTION AND PERSONALIZATION:

- Cultural adaptation of the contents (e.g., Ramadan, language, food, and culture).
- SMS timing and frequency should be customized.
- Consider patients’ circumstances and preferences. For example, using a wheelchair, having a caregiver for everyday activities, having Internet on their mobile phone, or whether or not they cook by themselves.
- SMSs should include the name of the patient.

ACCESIBILITY:

- Audible for people who cannot read SMSs due to different reasons (e.g., vision impaired, blind, or illiterate people).
- Different languages (Spanish, Catalan, Arabic, Chinese, English, and German).
- Easy to read, understandable, and short.
- Trusted source—the SMS sender must be well identified.

#### 3.2.3. PCPs Pointed Out Other Features and Technological Solutions They Would Like to Be Improved

Some professionals suggested that improving the electronic health records system, including a flagging system to remind them to perform specific tests and monitoring procedures, would also help them to improve the management of their diabetic patients.

*“If the system does not include feedback from patients, what would be the difference between this system and implementing a similar system in our own [registering] software? Alarms highlighting what should be done with each patient.” *

(Woman, 32, Doctor.)

Some PCPs focused more on the implications that the intervention would have for them than on what it would mean for their patients. As one doctor commented ‘*These messages, do they alert also health care professionals or, is it always the patient who, through these messages, has to go to make appointments?*’ (Woman, 24 years).

### 3.3. PCPs Views Raised Some Concerns and Limitations to Consider during the Design of the Intervention

#### 3.3.1. PCPs Have Mixed Feelings about Patient Empowerment

Although in general the messaging intervention was perceived as acceptable and useful, some of the participants expressed concerns about it, suggesting that the current system works well and that patients receive all they need. They expressed concerns about text messaging potentially causing patients to depend on their mobile phones and become overly reliant on the messaging system rather than being actively engaged in their care. This point of view largely came from PCPs who talked about “their patients” from a paternalistic perspective and were concerned about the possible negative consequences of the intervention. In fact, some of them proposed being involved in facilitating the intervention by being able to select the patients that should and should not receive the messages.

*“As a professional, I decide that I am going to offer you this intervention, and I know that this patient is going to receive messages and that suddenly he …will remind me when I have to do something.” *

(Man, 57, Doctor.)

Some professionals felt worried about being challenged by their patients, and not having enough training and communication skills to respond to new demands potentially arising from the messages. As one nurse said, ‘*My job is to inform patients about their disease and how to manage it, and about the consequences it may have for their health. So, if I don’t do my job, they will blame me.*’ (Man, 35 years).

Concerns were raised that patients may perceive that the messaging system involves a hidden agenda. Therefore, they raised the importance of obtaining informed consent to use their clinical data. They also stressed the importance of reassuring patients that their contact information would not be used to send advertisements and reminding them that they can withdraw from the program at any time.

*“This looks a bit like a TV ad. [patients] may think that the purpose of this system is to sell them something or manipulate them.”*

(Man, 61, Doctor.)

#### 3.3.2. Some Requirements Should Be Solved and Proved before Large-Scale Implementation of the Intervention

Professionals identified several barriers that need to be addressed before large-scale implementation of the messaging system. They noted that the system has structural requirements that must be considered when launching it: ‘*Health records are not up-to-date for a fair amount of our patients. In fact, people change their mobile number frequently*’ (Woman, 47 years), the doctor working in primary care management said. The same participant also stated that the ‘*SMSs content would need to be updated periodically*’, which constitutes an additional cost.

They perceived that the messaging system could result in an increased consumption of resources, such as blood tests or more frequent consultations. They agreed that resource use should be factored in by health decision makers prior to approving the rollout of the messaging system and suggested that a full health economic evaluation be conducted.

They hypothesized that if the intervention was cost-effective, it would save time and money for PCPs and the health system. They noted that reducing or delaying diabetes complications and hospital admissions are indirect measures of cost-effectiveness. They discussed the potential to use the intervention in other long-term conditions.

*“So, it could be a major reduction in workload that translates into lower costs, then more money available… and time, because time is money, which could be spent doing other things. In other words, it could have an impact on the organization of resources in primary care. How much? I do not know.”*

(Woman, 47, health decision maker.)

## 4. Discussion

### 4.1. Principal Findings

To our knowledge, this is the first qualitative study exploring the views and perspectives of PCPs on a text messaging intervention to support diabetes medication adherence in Spain. The intervention was perceived as potentially effective to support not only medication adherence, but also a number of additional aspects of DSM such as physical activity, a healthy diet, and diabetes complications. Participants highlighted the potential of DiabeText to increase disease awareness in people with T2D, promote DSM, and constitute an additional service to complement clinical appointments and follow-up. PCPs also highlighted that they need to be actively involved in the development and implementation of the intervention because they know what people with T2D need in terms of information and the clinical objectives which would be addressed through the intervention. Finally, we also observed some concerns about the intervention. While some PCPs perceived the intervention as a tool for patient empowerment, others felt that the intervention may cause dependence on it. Additionally, the intervention was perceived to have important implications in terms of resource use. Obtaining solid evidence about its effectiveness and cost-effectiveness was therefore identified as a key prerequisite for its large-scale implementation.

### 4.2. Comparison with Prior Work

A recent systematic review examined health workers’ perceptions and experiences of using mHealth technologies to deliver primary healthcare services [24]. Although this qualitative review targeted a wide range of mHealth technologies and included studies from low- or middle-income countries, some conclusions were in accordance with ours. For example, professionals valued mHealth interventions as long as they were not time-consuming. As in our study, the systematic review observed that mHealth interventions may lead to new forms of engagement and relationships between patients and health professionals (HPs). Additional common findings between our study and the systematic review were that health professionals supported the use of electronic health records to deliver personalized healthcare but were concerned about compromising patients’ personal data and that HPs were concerned about accessibility and equity issues that may arise from differential phone usage levels, language, and poverty issues. In line with our findings, previous studies identified patient barriers to mobile phone interventions as being older, having a low educational level, and having a lack of technological skills [25,26,27].

Our findings largely resonate with those from another recent qualitative systematic review that examined healthcare professionals’ perspectives on technology-assisted diabetes self-management education [28]. Although the authors concluded that HPs show high levels of acceptance towards technologies for DSM education, HPs identified several concerns, such as the difficulty of integrating those technologies into their workflows due to their independent nature and the lack of time to conduct and document their use. Additionally, HPs were concerned about the mismatch between the complexity of the content and the target audience’s health literacy levels, where only those with better health literacy would benefit from the intervention. Lastly, HPs noted that if there was variability in the information provided from the system and other sources because of a lack of standardization, then patients would be deterred from using them because of a lack of reliability.

In a cross-sectional study about HPs opinions concerning a diabetes patient web portal [26], the authors observed that, although providers were in favor of the intervention, and they agreed that the web page improved patients’ knowledge of diabetes and their quality of care, they would not recommend it to all patients. HPs considered that test results and clinical notes should not always be shared with patients, which is in line with the skepticism and lack of confidence in patient empowerment shown by some of the participants in our study.

Some other HPs’ perspectives about technological interventions addressing the healthcare education of patients with cardiovascular diseases have been described [25,27]. Among them, monitoring of emotional status and health measures has been proposed for the improvement of patients’ self-awareness in terms of their disease management [25]. Personalization of the intervention and the use of a patient-centered approach was also advocated by HPs to facilitate optimal engagement [27].

### 4.3. Implications

The results obtained in this study will be used to guide specific aspects of the design and development of the DiabeText intervention (and potentially also other similar mHealth interventions). First, we will ensure that PCPs are adequately informed about this research study and that they feel they are part of it (creating a sense of ownership). To that end, we will develop a communication strategy targeted at PCPs, which will be implemented before and during the study period. Second, we will ensure that the interventions do not impose an increased workload on PCPs. Tasks such as patient recruitment or blood test screenings will be carried out by members of the research team rather than by PCPs. Third, for messages to be perceived as trustworthy by PCPs, we will ensure they are evidence based and clearly aligned with current clinical guidelines. We will involve nominated PCPs in the development of the messages. Finally, our post-trial dissemination strategy will include results of the cost-effectiveness of the DiabeText intervention—as in this qualitative study, cost-effectiveness was identified as an important feature for decision makers considering rolling out the DiabeText intervention.

### 4.4. Strengths and Limitations

An important strength of this study is its methodological rigor. The study meets the main trustworthiness criteria: credibility (multiple coders, triangulation), dependability (different focus groups conducted at different HCs and on different days pointed to similar results), transferability (the provision of information about the context and participants suggests results may be transferred to other groups which want to introduce a mHealth intervention in the primary care setting), conformability (measures were taken in data collection and analysis to ensure that all researchers engaged in reflexivity and in-depth discussion at the analysis stage which allowed us to tease out and agree on the final analysis), and authenticity (all data were included in the analysis and dissenting views were considered and presented) [29]. Additionally, we interviewed physicians and nurses from different primary health centers, with different structures, workloads, and attending patients with different characteristics; and included individual interviews with one endocrinologist, one community pharmacist, and one health decision maker. This resulted in successfully collecting a wide range of views and perspectives from multiple stakeholders—a key aspect of informing the development of complex interventions [20] such as DiabeText.

The limitations of the study are common to formative research studies. First, we gathered the views of staff in existing PCP networks with experience and training in diabetes care. Staff who chose not to participate in this study may have held different views than those expressed by the participants. Although we think this is not a major limitation (our data were widely varied and included views that were supportive and critical of the intervention), when developing the intervention, it will be important to gather the views of PCPs who are outside of these networks. Second, in some cases, some focus group participants may find it difficult to express their views, and this may be particularly true when: (1) They know each other; (2) When predefined power structures within groups are present. This is particularly relevant in our study because our focus groups gathered doctors and nurses from the same centers. However, efforts were made to include all participants and elicit their views. The addition of individual interviews also allowed staff to express controversial or minority views. Third, the views of patients were not incorporated into this analysis. However, these data were gathered and is available elsewhere [21]. It is nonetheless important to voice the views of PCPs separately as they are key stakeholders in this intervention. Finally, it is worth noting that this study was conducted prior to the onset of the current COVID-19 pandemic. The views and perspectives of the primary care professionals may have changed as a result of the reorganization of health services imposed since then. Therefore, replicating our study in this new context would be helpful to explore the extent to which providers’ perspectives remain unchanged and are still applicable.

## 5. Conclusions

This qualitative study investigated the potential barriers and enablers of the acceptance and perceived utility of an mHealth intervention for diabetes care by PCPs in Spain. Three main themes were developed from the data which form a platform for future research: (1) This type of novel intervention has the potential to support DSM in patients with T2D; (2) PCPs may have an important role in all the steps required to implement a technological system based on mobile texting to patients; (3) Before implementation, cost-effectiveness should be proven to gain the trust of PCPs and other health management figures. Our findings could be used to inform the design of new text messaging interventions as well as other more complex digital therapeutic solutions for diabetes and other chronic conditions.

## Supplementary Materials

The following are available online at https://www.mdpi.com/article/10.3390/ijerph19074237/s1, Table S1: Extended list of quotations from participants associated to each theme and subthemes identified.
