# Supplementary material for: Perspectives and Views of Primary Care Professionals Regarding DiabeText, a New mHealth Intervention to Support Adherence to Antidiabetic Medication in Spain: A Qualitative Study
Source: Int J Environ Res Public Health. 2022 Apr 1;19(7):4237. doi: 10.3390/ijerph19074237 (PMC8999018; doi:10.3390/ijerph19074237)
Supplement: Supplementary file 1 [file ijerph-19-04237-s001.zip › ijerph-1611559-supplementary.pdf]

Table S1. Extended list of quotations from participants associated to each theme and subthemes identified.

**Theme 1. The intervention has the potential to effectively support the provision of diabetes care.**	
**Subthemes**	**Quotations**
DiabeText could increase awareness of the disease and adherence to treatment	"I think we should use these technologies if we have them because people use them more and more" (Man, 40, Doctor).<br><br>"The messages could be used as a means to reach all those diabetic patients who are not aware that they are diabetic. For example, I have patients who take metformin, but still, they are not aware that they have diabetes" (Woman, 46, Doctor).<br><br>"I think that for us, it [the intervention] could be a positive thing, in the sense that patients would perceive that the health system takes care of them, their treatment is taken into account, and that, therefore, they are taken care of, and one way to show it is by providing reminders about how they have to take their medication when to take the pills, and perhaps additional information to reinforce adherence or lifestyles. I think that everything – taking into account the person and monitoring them – has a positive impact that helps to improve adherence. And if adherence improves, control improves, and if control improves, the system is more sustainable". (Woman, 48, Nurse.)
DiabeText could support for medical consultations	"We have lots of data, indicators, but we have less and less connection with our patients. Every time we diagnose more, but we do worse at controls and follow-ups. I think the key thing is to spend time with our patients". (Man, 47, Doctor.)<br><br>"It happens to us many times: we can shake hands with the patient, but we are not able to look at their feet". (Man, 47, Doctor.)<br><br>"The issue is that you have to investigate in five different windows where the five different registries of the diabetes complications; and I think that it ends up complicating and discouraging the registry, even to review it". (Woman, 32, Doctor.)<br><br>"If the message is consistent with the scientific evidence and with the same message that we are giving, I imagine that, in some way, these messages will have to be developed in a consensual way, by a group of professionals, based on the latest evidence, and so on." (Woman, 52, Nurse.)

	"I think that, if it were to be implemented…outside of …the study framework... obviously, the contents will have to be kept updated as time goes by. Then, primary care professionals, who are active and knowledgeable, could contribute to modifying the messages, incorporating the views of the professionals who have direct contact with patients…so that their [patients"] opinion is taken into account." (Woman, 47, Doctor.)<br><br>"We will explain to the patient that they will receive messages to guide them through the diabetes care management and if they have any doubt they can come to us for solving it" (Woman, 33, Nurse.)<br><br>"The most difficult thing for us is to inculcate in them a change of habits, diet, and exercise." (Man, 40, Doctor).
DiabeText could support to promote DSM	"The mHealth intervention could take away from us the paternalism role that we are playing, but we are desperate not to act like that" (Woman, 58, Nurse.)<br><br>"The patients are ones who should be aware of their situation, their illness, and they should be the ones saying "I need to have my feet checked", for example." (Woman, 30, Nurse.)<br><br>"[the messaging intervention] is especially useful for this type of patients we usually do not reach - the ones not coming to appointments, who are the ones less well-controlled." (Man, 57, Doctor.).<br><br>"[the messaging intervention] is good to receive it at home, where you are calm and can analyze everything the doctor told you during the consultation. Patients say "yes, yes, I under-stand, yes, yes", but actually they do not understand anything." (Woman, 47, Doctor.)<br><br>"I believe that, for example, to a patient who is not well controlled, who you may have already visited, probably, (…) it is okay to send reinforcement through this message system. So, you send him a personalized message, remembering very specific aspects of what was discussed during the last visit, and this can reinforce what you have already told him." (Men, 57, Doctor.)
**Theme 2. Involving health professionals in the intervention would facilitate its design and implementation.**	
**Subthemes**	**Quotations**

PCPs highlight the importance of being part of the project for its success	"Primary care professionals could shape the messages, incorporating their point of view (…). Professionals could contribute to incorporate the patients" views on the messages, offering a means of communication so that their opinion is taken into account" (Woman, 47, Doctor.).<br><br>"If we make an extraordinary product, but we do not know how to advertise it, we will not have a high market share, we will even have to close the company." (Man, 47, Doctor.)<br><br>"The primary care team has to receive the recognition they deserve. The messages should not replace their work" (Woman, 30, Nurse.).<br><br>"Yes, there are some difficulties with the implementation of the intervention. For example, knowing who manages the messages and their appropriateness to the patient. Logically, if messages are personalized, professionals have to be aware of the situation. It is important that the professional does not feel invaded, that is, things are done to their patients without knowing it." (Man, 58, Doctor.)
PCPs pointed out other features and technological solutions they would like to be improved	"These messages, do they alert also health care professionals or, is it always the patient who, through these messages, has to go to make appointments?" (Woman, 24, Doctor.)<br><br>"If the system does not include feedback from patients, what would be the difference between this system and implementing a similar system in our own [registering] software? Alarms highlighting what should be done with each patient." (Woman, 32, Doctor.)
**Theme 3. PCPs views raised some concerns and limitation to consider during the design of the intervention.**	
**Subthemes**	**Quotations**
PCPs have mixed feelings about patient empowerment	"As a professional, I decide that I am going to offer you this intervention, and I know that this patient is going to receive messages and that suddenly he …will remind me when I have to do something." (Man, 57, Doctor.)<br><br>"My job is to inform patients about their disease and how to manage it, and about the consequences it may have for their health. So, if I don't do my job, they will blame me." (Man, 35, Nurse).<br><br>"I think that the professional should be able to alert the research team to activate the sending of messages. In the first place, for T2D people who you think will be

	interested and then for people who have bad control. The well-controlled may not be the first to start sending messages to.” (Woman, 59, Nurse.)<br><br>“This looks a bit like a TV ad. [patients] may think that the purpose of this system is to sell them something or manipulate them.” (Man, 61, Doctor.)
Some requirements should be solved and proved before large-scale implementation of the intervention	“Health records are not up-to-date for a fair amount of our patients. In fact, people change their mobile number frequently” (Woman, 47, Health decision-maker)<br><br>“SMSs content would need to be updated periodically” (Woman, 47, Health decision-maker)<br><br>“Before implementing it, have a good pilot study done to see that the algorithms work and that there are no failures. If this really benefits the patient, the patient will wait for it. If it benefits healthcare professionals, they will empower it and support it.” (Man, 58, Doctor.)<br><br>“So, it could be a major reduction in workload that translates into lower costs, then more money available... and time, because time is money, which could be spent doing other things. In other words, it could have an impact on the organization of resources in primary care. How much? I do not know.” (Woman, 47, Health decision-maker.)<br><br>“These types of projects, if they do work, should be exported to other pathologies to benefit the maximum number of patients and, therefore, this positive impact would increase.” (Woman, 48, Nurse.)

DSM: diabetes self-management; PCPs: Primary Care Professionals.
